# Biodegradation of PBSA Films by Elite *Aspergillus* Isolates and Farmland Soil

**DOI:** 10.3390/polym14071320

**Published:** 2022-03-24

**Authors:** Hsiao-Lin Chien, Yi-Ting Tsai, Wei-Sung Tseng, Jin-An Wu, Shin-Liang Kuo, Sheng-Lung Chang, Shu-Jiuan Huang, Chi-Te Liu

**Affiliations:** 1 Institute of Biotechnology, National Taiwan University, Taipei 106, Taiwan; d99642014@ntu.edu.tw (H.-L.C.); r08642004@ntu.edu.tw (Y.-T.T.); t3885863412@gmail.com (W.-S.T.); 2 Material and Chemical Research Laboratories, Industrial Technology Research Institute, Hsinchu 300, Taiwan; itriA50018@itri.org.tw (J.-A.W.); slkuo@itri.org.tw (S.-L.K.); itria00534@itri.org.tw (S.-L.C.); amyhuang@itri.org.tw (S.-J.H.); 3 Department of Agricultural Chemistry, National Taiwan University, Taipei 106, Taiwan; 4 Agricultural Biotechnology Research Center, Academia Sinica, Taipei 115, Taiwan

**Keywords:** polybutylene succinate-co-adipate (PBSA), biodegradation, *Aspergillus*, phytotoxicity, ecotoxicity, lipolytic enzyme

## Abstract

Plastic films are widely used in current agricultural practices; however, most mulch films used are discarded and buried in the land after harvest, having adverse environmental impacts. To solve this environmental problem, the demand for biodegradable mulch has been increasing in recent years. Polybutylene succinate-co-adipate (PBSA) is a biodegradable polymer with good ductility and can be used for packaging and mulching. In this study, we isolated two elite fungal strains for PBSA degradation from farmlands, i.e., *Aspergillus fumigatus* L30 and *Aspergillus terreus* HC, and the latter showed better degradation ability than the former. It is noteworthy that biodegradation of PBSA by *A. terreus* is reported for the first time, which revealed unique characteristics. In the soil burial test, even the soil with relatively poor degradation ability could be improved by the addition of elite fungal mycelia. In substrate specificity analyses of soil samples, PBSA could induce the synthesis of lipolytic enzymes of indigenous microbes to degrade substrates with medium and long carbon chains in soil. Furthermore, PBSA residues or fungal mycelia supplementation in soils had no adverse effect on the seed germination rate, seedling growth, or mature plant weight of the test green leafy vegetable. Taken together, the results of this study not only advance our understanding of the biodegradation of PBSA films by filamentous fungi but also provide insight into improving the efficiency of biodegradation in soil environments.

## 1. Introduction

Plastics are widely used in our daily life and industries worldwide. The materials of conventional plastics are mostly derived from nonrenewable fossil fuels. The treatment of plastic wastes is mainly divided into three categories: incineration, recycling, and landfill burying [1]. However, the recycling rate of plastic is relatively low at approximately 9%, and almost 60% of plastic waste is discarded in landfills or natural environments [2]. Although marine plastic pollution is an environmental issue that has attracted much attention in recent years, the amount of plastic waste discarded and retained in the terrestrial environment is estimated to be 4–23 times that released into the ocean [3].

The main sources of terrestrial plastics include sewage sludge (i.e., application as fertilizer for agricultural lands), controlled-release fertilizers, and agricultural plastics (e.g., polytunnels and plastic mulches) [3]. In particular, the impact of agricultural mulch film on the environment is usually underestimated. Mulch film can effectively increase the surface temperature of farmland, maintain soil moisture, prevent soil loss, inhibit weed growth, promote seed germination, exceed seedling growth, and indirectly increase crop yields [4,5]. Currently, the majority of commercially available mulch film on global markets is prepared with polyethylene (PE) and low-density polyethylene (LDPE) (Transparency Market Research, 2019) [4]. More than 2 million tons of mulch film are used globally every year, most of which becomes mulch film waste within one to two years [6]. Mulch films are damaged to varying degrees due to weathering and photocatalytic degradation during crop cultivation [7]. After use, farmers need to pay additional expenses for mulch film waste disposal. Most of the mulch films used are incinerated or buried. In addition, conventional mulch films, such as LDPE and other raw materials buried in farmland, affect soil drainage and crop absorption of water because of their low degradation rate, and these films also pollute soil and water environments by the release of microplastics [3,8].

Based on the consideration of sustainable environmental development and reduction in the health hazards of plastics, in addition to imposing taxes on the production of traditional nondegradable plastic products or directly imposing use bans, many countries also actively encourage the development and use of biodegradable plastic materials [4]. Taking biodegradable plastic mulch films as an example, in recent years, the demand for these films has continued to increase. The estimated output value of plastic mulch films will reach USD 5 billion in 2024 (Zion Market Research, 2018, New York, NY, USA), and these films will have a compound annual growth rate (CAGR) of 9.4% (2017–2023) (Prescient & Strategic Intelligence Private Limited, 2018, USA). Common biodegradable mulch film materials can be divided into three categories: polysaccharides, aliphatic polyesters, and aromatic polyesters, including polylactic acid (PLA), polyhydroxybutyrate (PHB), starch blends, cellulose, polybutylene adipate terephthalate (PBAT), and polybutylene succinate (PBS) [9]. PBAT mulch films are commonly used in agriculture because of their strong mechanical properties, and PLA is often blended with other polymers because of its strength and low cost, but these compounds need to be decomposed by microorganisms in a relatively high-temperature or high-humidity environment [4]. PHB is produced by microorganisms, but its use is limited because of its brittleness, crystalline characteristics, and high production cost [4,9]. PBS is often copolymerized with other polymers or forms copolymers such as polybutylene succinate-co-adipate (PBSA) to reduce its crystallinity and improve decomposition efficiency [4].

PBSA is a biodegradable, aliphatic copolymer synthesized by 1,4-butanediol (BDO) with succinic acid (SA) and adipic acid [10,11]. PBS is produced by SA and BDO, and PBA is produced by BDO and AA [12]. PBSA possesses high flexibility, processability, excellent impact strength, and good thermal and chemical resistance [13,14]. The semicrystalline characteristic of PBSA makes this material relatively easy to biodegrade and is applied to packaging and mulch film production [12,15]. In a previous study, Yamamoto-Tamura and colleagues collected cultivated field soil samples from 11 sites in Japan, where PBSA films (2 × 2 cm^2^, thickness: 20 µm) were buried on a laboratory scale. According to their results, the degradation rates of PBSA films ranged from 1.4% to 95.9% after four weeks of incubation [16].

In the depolymerization steps of biodegradation, microbes secrete a variety of extracellular enzymes to turn polymer chains into oligomers or monomers [17]. The hydrolytic enzymes working on carboxyl ester bonds in lipids are called lipolytic enzymes, which include carboxylesterases and lipases [18]. Lipases (EC 3.1.1.3, triacylglycerol hydrolases) and esterases (EC3.1.1.1, carboxyl ester hydrolases) are the major enzymes for the degradation of polyester-based biodegradable plastics [19]. Lipase has been considered as one of the good candidate enzymes for PBSA degradation [20]. Several studies have reported that fungal lipases could degrade PBSA. For example, the lipases secreted from *Pseudozyma antarctica* JCM 10317, *Rhizopus niveus*, *Rhizopus oryzae*, *Candida cylindracea*, and *Rhizopus delemar* have been demonstrated [21,22,23]. Similarly, esterase also plays an important role in the degradation of bioplastics by hydrolyzing polymer materials [24]. The esterase activity of *Leptothrix* sp. was found to be increased along with PBSA degradation and microbial growth [25]. The number average molecular weight [26] of PBSA was decreased from 5.9 × 10^4^ to 1.5 × 10^4^ by treating *Pseudozyma antarctica* esterase [27]. In addition, the recombinant protein (FSC) derived from *Fusarium solani* showed strong PBSA degradation activity (179.6 U/mg), with relatively high esterase activity (287 U/mg) [28].

According to the literature, many fungal strains belonging to the genus *Aspergillus* possess the potential for bioplastic degradation. For example, Merugu and colleagues isolated seven *Aspergillus* species from soil (*A. niger*, *A. flavus*, *A. ochraceus*, *A. nidulans*, *A. terreus*, *A. fumigatus*, and *A. parasiticus*) showed PHB degradability [29]. *Aspergillus* is a versatile plastic degrader. *A. fumigatus* is reported to be able to degrade PHB, polysuccinic acid [30], PBS, polycaprolactone, PCL, and PLA [31].

The aim of this study is to select elite microorganisms that are able to be applied in the soil environments for biodegradation of PBSA mulch film. We reported two PBSA-degrading *Aspergillus* strains (*A. fumigatus* L30 and *A. terreus* HC) isolated from farmland soils. Their PBSA-degrading activity was assessed under medium culture conditions. Additionally, we evaluated the biodegradability of PBSA films buried in farmland soils. We also determined the activities of the lipolytic enzymes of the two isolates and those of the soil samples for PBSA degradation. After degradation of the PBSA films in soil, we conducted a phytotoxicity assay to evaluate any harmful effects of the soil containing PBSA residues on the growth of seedlings and adult plants of Chinese cabbage.

## 2. Materials and Methods

### 2.1. Plastic Material

PBSA (number average molecular weight, Mn = 51,899 Daltons; weight average molecular weight, Mw = 77,951 Daltons) particles and films (thickness: 50 μm) were provided by the Industrial Technology Research Institute (ITRI). The constituent ratios of SA, AA, and BDO for the PBSA film were 37%, 13%, and 49.9%, respectively. The film was cut into 2.5 cm × 5.0 cm or 5.0 cm × 5.0 cm fragments for degradation assessment under liquid conditions or for the soil burial degradation test, respectively.

### 2.2. Screening and Isolation of PBSA-Degrading Microorganisms from Soils

The test soils were collected from the following 8 sites (9 samples in total) in Taiwan: Four samples were collected from the bank of the Xindian River topsoil (XDR-A (24°59′09.7″ N 121°31′37.4″ E), XDR-B (24°59′21.3″ N 121°31′51.0″ E), XDR-C (24°59′30.5” N 121°31′56.5″ E), and XDR-D (24°59′30.9” N 121°31′56.4” E)); two samples were collected from a composting yard at National Taiwan University (25°00′57.9″ N 121°32′31.4″ E) (NTUCS-S collected from the surface (0–20 cm depth), and NTUCS-D collected at a 50 cm depth in the compost soil); two samples were collected from a rice paddy field topsoil in Xinfeng Township, Hsinchu County (HCP-A (24°55′47.9″ N 121°00′34.4″ E) and HCP-B (24°55′45.2″ N 121°00′33.8″ E)); and the final sample was collected from a tomato farm topsoil located in Xiushui Township, Changhua County (CHT (24°01′08.8” N 120°31′02.5” E)). All the soil samples were sieved through 3 mm mesh before use.

We applied a rapid screening platform (i.e., clear zone method) to select elite microorganisms for PBSA degradation, as proposed by [32]. Ten grams of PBSA plastic particles were dissolved in 40 mL of chloroform (Sigma–Aldrich, Merck & Co., Taipei, Taiwan), and the solution was emulsified with 1 g/L commercial detergent (PAOS^®^, Nice Co., Chiayi County, Taiwan), mixed with 200–300 mL of carbon-free basal medium (0.7 g/L KH_2_ PO_4_, 0.7 g/L MgSO_4_·7 H_2_ O, 1 g/L NH_4_ NO_3_, 0.005 g/L NaCl, 0.002 g/L FeSO_4_·7 H_2_ O, 0.002 g/L ZnSO_4_·7 H_2_ O, and 0.001 g/L MnSO_4_·H_2_ O) [33,34]. The plastic solution was homogenized by a sonicator for at least 15 min, and then the volume was brought to 1 L with a carbon-free medium. Volatile chloroform was removed in a fume hood overnight, and the pH was adjusted to 7.0. Then, 1.2% agar was added and autoclaved (121 °C, 20 min) to prepare PBSA agar plates [34].

One gram of individual soil sample was dissolved in 5 mL of distilled water and shaken at 100 rpm for 15 min at room temperature. The respective soil suspension (100 µL) was spread over the surface of the plastic solid medium by glass beads and incubated at 25, 30, or 37 °C for 9 days. When clear zones were formed on the plates, a single colony of individual PBSA-degrading microorganisms was isolated by the streaking plate method. According to the morphological characteristics of the isolates with hyphae and spores, we speculated that these isolates were fungi.

### 2.3. Phylogenetic Analysis of ITS Gene Sequences

Genomic DNA from the PBSA-degrading fungal strains was isolated by a Presto™ Mini gDNA kit (Geneaid Biotech Ltd., Taipei, Taiwan). The primer sets ITS4 (5′-TCC TCC GCT TAT TGA TATGC-3′), and ITS5 (5′-GGA AGT AAA AGT CGT AAC AAG G-3′) were used to amplify the ITS region of fungi [35,36]. PCR products of approximately 400 bp were expected and sequenced through paired-end sequencing. Amplicons were Sanger sequenced at the Center of Biotechnology at National Taiwan University. The sequencing results were edited with BioEdit 7.2.6 software [37]. The obtained DNA sequences were identified against the GenBank database using the Basic Local Alignment Search Tool (BLAST) (https://blast.ncbi.nlm.nih.gov/Blast.cgi/, accessed on 20 July 2021) of the National Center for Biotechnology Information (NCBI). The phylogenetic tree was generated by the neighbor-joining method (1000 bootstrap repeats) on version X Molecular Evolutionary Genetics Analysis (MEGA) software (The Pennsylvania State University, State College, PA, USA).

### 2.4. Determination of the PBSA Film Degradation Ability of Isolated Fungal Strains

PBSA film (size: 2.5 × 5.0 cm^2^; thickness: 50 µm) was sterilized with 6% sodium hypochlorite (Sigma–Aldrich Co., St. Louis, MO, USA) and 70% ethanol for 10 min, washed twice with sterile water, and then kept in the carbon-free medium before use. The spore suspension for inoculation was prepared by culturing the respective elite strain (*Aspergillus fumigatus* L30; *Aspergillus terreus* HC) and the reference strain for PBSA-degradation *Aspergillus oryzae* RIB40 (ATCC 42149) cultured on potato dextrose agar (PDA) for 7–10 days. The spores were collected with sterile distilled water containing 0.05% Tween 20 (Bioman Scientific Co., Ltd., Taipei, Taiwan). The final concentration of the inoculated spore suspension was 2.5 × 10^6^ spores/mL, and the spores were cultured with 12 pieces of PBSA film in 100 mL of the carbon-free medium at 30 °C and 80 rpm. The plastic films were stirred gently every week to prevent uneven decomposition caused by film overlap. Three pieces of the films were removed from the flask after 14 and 30 days of incubation, and the attached mycelia were removed from the surface of the films. After washing the films carefully with distilled water to remove the attached hyphae, the dry weights were determined after drying in a 60 °C oven overnight. All tests were performed in at least three independent biological replicates.

The effect of the addition or replacement of fresh medium for long-term (60 days) degradation of PBSA film was also conducted with *A. fumigatus* L30 and *A. terreus* HC, respectively. The preparation for PBSA film degradation by the respective elite fungal culture was the same as mentioned above. After 30 days of incubation, 30 mL of fresh carbon-free basal medium was added to the culture fluid, or the total culture fluid (100 mL) was removed carefully by pipette and replaced with an equal amount of fresh basal medium. The attached mycelia were removed from the surface of PBSA films after 30 and 60 days of incubation, and their weights were determined.

The degree of degradation was evaluated by weight loss (WL) using the following modified equation [38]: Plastic remaining weight (%) =100% − (Wi − Wt)/Wi × 100%, where Wi is the initial weight of the sample, and Wt is the weight after the incubation time.

### 2.5. Scanning Electron Microscopic Analysis

To observe the plastic surface erosion or decomposition after microbial degradation, we used scanning electron microscopy [39] (Jeol, JSM-6510, Tokyo, Japan). The SEM sample preparation protocol followed that of the Joint Center for Instruments and Researchers, College of Bio-Resources and Agriculture, NTU. Plastic films cultured with *A. fumigatus* L30 or *A. terreus* HC for 30 days were removed from the flask, and each film was cut into 2 pieces and divided into washed and unwashed groups. For the washed group, the plastic film was washed with distilled water to remove the mycelia attached to the plastic surface. The plastic films were immediately soaked in 2.5% (*w*/*v*) glutaraldehyde (Sigma–Aldrich Co.) at 4 °C and then shaken overnight for cell fixation. The fixed films were washed with 0.1 M sodium phosphate buffer (pH = 7.3) and postfixed in 1% osmium tetroxide (*w*/*v*) in an ice bath for 1 h. The samples were dehydrated gradually with different concentrations of ethanol (30%, 50%, 70%, 85%, 90%, 95%, 100%) for 60 min at each concentration, except for 70% and 100% ethanol for which overnight dehydration was conducted. The dehydrated samples were placed in a critical point dryer for critical spot drying, and subsequently, the samples were coated with gold and observed under a scanning electron microscope (Jeol, JSM-6510) [40].

### 2.6. NMR Analysis

For NMR sample preparation, PBSA plastic films after 30 days of degradation (0.1~0.05 g) were dissolved in 3 mL of deuterated chloroform (CDCl_3_). The 1 H NMR analysis was conducted by the Industrial Technology Research Institute/Material and Chemical Laboratories (ITRI/MCL) with a Varian INOVA 500 NMR spectrometer (Varian Medical Systems, Inc., Palo Alto, CA, USA). The 1 H NMR spectra were recorded at 25 °C (128 scans, 1 s relaxation delay), and used tetramethylsilane (TMS) was employed as an internal reference for the reported chemical shifts.

### 2.7. Soil Burial Tests of PBSA Plastic Films

To verify the degradation of the PBSA plastic films in the general soil environment, we conducted a soil burial test following a previous method with some modifications [10]. The soil was collected from the experimental farm of the College of Bioresources and Agriculture, National Taiwan University. The effect of temperature on biodegradation was also evaluated by sampling soil in September (26.3 °C~32.1 °C, avg. 27.8 °C) and December (15 °C~24.7 °C, avg. 18.1 °C). The soil samples were sieved in advance through a 3 mm stainless steel sifting screen and transferred to a sterilized plastic box (18.1 (L) × 12.8 (W) × 6.8 (D) cm^3^, HPL815 M, Lock & Lock, Hana Cobi Plastic Co, Ltd., Seoul, Korea).

Three PBSA film samples (size: 5.0 × 5.0 cm^2^, thickness: 50 μm, initial weight: approximately 0.2 g) were sandwiched between two layers of soil (each layer was 1 cm thick and weighed 200 g, as shown in Figure 5A). Four treatments were conducted, including soil, sterile soil, and soil supplemented with low/high doses of *A. terreus* HC fungal hyphae. In the sterile soil group, the soil used was sterilized by an autoclave (121 °C, 40 min) before the trial. For the low- and high-dose groups, 10 mL (cell dry weight approximately 0.04 g) or 50 mL (cell dry weight approximately 0.16 g) of mycelial suspension were added, respectively. Six holes were drilled in the lid of the respective box for aeration, and the boxes were placed in an incubator at 25 ± 1 °C under moisture-controlled conditions.

For all treatments, water was added until the soil water content reached 50% of the maximum water holding capacity (WHC) according to the EN17033 and OECD Guideline (https://www.iso.org/obp/ui/#iso:std:iso:23517:ed-1:v1:en/, accessed on 25 December 2021). One piece of film was collected from each box weekly and weighed. Each plastic film was brushed softly and washed with distilled water several times to remove the attached soil and microorganisms. The washed plastic samples were placed in a Petri dish and dried overnight in an oven at 60 °C before weighing. After weekly sampling, the whole box was weighed and refilled with distilled water to maintain a 50% WHC water content. The degree of degradation was evaluated by WL as described previously.

### 2.8. Analysis of Lipolytic Enzyme Activities in Culture Supernatant

The lipolytic enzyme activity analytical procedure was modified based on a previous study [41]. *A. terreus* HC was cultured in basal medium with PBSA plastic film pieces (2.5 cm × 5.0 cm, 0.1 g) at 30 °C for 60 days. After 30 days of incubation, 30 mL of fresh carbon-free basal medium was added to the culture broth and incubated for an additional 30 days. After 60 days of incubation, all the PBSA film pieces were taken from the broth. The total amount of the culture broth was collected and filtered through Qualitative Filter Paper NO. 1 (TOYO ADVANTEC) and a 0.22 µm filter to remove residual hyphae and the visible suspended matter, and a centrifugal concentrator (molecular weight cutoff (MWCO) 10 kDa, Sartorius) was used at 5000× *g* at 4 °C to condense the culture fluid. Then, the sample was dialyzed with a cellulose membrane (EIDIA, catalog number: UC27-32-100, 14,000 Dalton MWCO) in 0.1 M Tris-HCl. The dialyzed sample and crude protein suspension were stored at −20 °C for further enzyme activity analysis.

The lipolytic enzyme activity assay was modified based on previous studies [39,42]. *p*-nitrophenyl esters are usually used as substrates to measure lipase/esterase activity [43]. Thus, 900 µL of crude protein suspension was added to 100 μL of 10 mM 4-nitrophenyl acetate (C2), 4-nitrophenyl butyrate (C4), 4-nitrophenyl caprylate (C8), 4-nitrophenyl decanoate (C10), 4-nitrophenyl dodecanoate (C12), or 4-nitrophenyl palmitate (C16) (Sigma–Aldrich Co.), which were dissolved in DMSO as the substrate. The solution was incubated at 30 °C for 30 min. Fluorescence analysis of the chromogenic pNP products derived from the hydrolyzed *p*-nitrophenyl substrates was conducted by a microplate reader (Thermo Scientific™ Varioskan™ LUX, VLBL00 D0, Thermo Fisher Scientific Inc., Taipei, Taiwan) at a wavelength of 405 nm. For the preparation of the pNP calibration standard curve, 0, 25, 50, 75, 100, or 125 μg of pNP was dissolved in DMSO buffer and incubated at 30 °C for 30 min. We used a microplate reader to measure the optical density at 405 nm (O.D_405_). The enzyme activity unit (Unit) is expressed as the production of 1 µmole of nitrophenol product per minute per mL of crude protein.

### 2.9. Analysis of Lipolytic Enzyme Activities in Soil

The soil samples used in this assay were those containing PBSA residue after 30 days of degradation as described in the above section titled “*Section 2.7*”. To analyze the lipolytic enzyme activities of the soil containing decomposed PBSA films, we conducted the following procedures with slight modifications [44]. Individual soil samples (0.05 g) were suspended in 1 mL of 100 mM NaH_2_ PO_4_/NaOH buffer (pH 7.25) and mixed well at 30 °C and 100 rpm for 30 min. Then, 80 µL of 25 mM nitrophenyl esters with different lengths of carbon substrates (i.e., 4-nitrophenyl acetate (C2), 4-nitrophenyl butyrate (C4), 4-nitrophenyl caprylate (C8), 4-nitrophenyl decanoate (C10), 4-nitrophenyl dodecanoate (C12), or 4-nitrophenyl palmitate (C16) (Sigma–Aldrich Co.) were dissolved individually in isopropanol (Sigma–Aldrich Co.) and mixed well at 30 °C and 100 rpm for 30 min. To measure the pNP released from individual substrates, we prepared a buffer control without soil suspension. A sample of soil background without pNP substrates was also prepared as a control. To stop the reaction, the solutions were cooled on ice for 10 min. Afterward, each solution was centrifuged at 3000× *g* and 4 °C for 10 min. Then, 100 µL of supernatant was collected via pipette, and the OD_405_ was determined by a microplate reader (Thermo Scientific™ Varioskan™ LUX, VLBL00 D0, Taipei, Taiwan) to measure pNP products derived from the hydrolyzed *p*-nitrophenyl substrates. To prepare the pNP calibration standard curve, 0, 25, 50, 75, 100, or 125 μg of pNP was dissolved in 100 mM NaH_2_PO_4_/NaOH buffer and incubated at 30 °C for 30 min. The standard contents were cooled on ice for 10 min and centrifuged at 2000× *g* and 4 °C for 10 min. A microplate reader was used to measure the OD value of each soil sample.

### 2.10. Phytotoxicity Assessment of the Soil Containing PBSA Residues

This experiment followed a previous method with some modifications [45]. The soil was collected from the experimental farm of National Taiwan University and sieved through a 3 mm stainless steel sifting screen. Four pieces of PBSA films were buried in the device utilized for the soil burial test for phytotoxicity assessment. The total weight of plastic films (i.e., approximately 0.8 g) was a mass percentage of approximately 0.2% of the total soil weight (i.e., 400 g). The total weight of the soil system was determined every week, and distilled water was replenished to maintain the water content as described above. After 30 days of incubation, soil containing PBSA residue was filled into 128 holes of a plug tray (3 × 3 × 3 cm^3^ for each hole). Each group contained 64 seeds of Chinese cabbage (*Brassica rapa* L. ssp. *chinensis* var. Maruha), which were sown on the surface of the soil to observe germination. After the seeds were germinated and cultivated until the seedlings with two true leaves appeared (~8 days of cultivation), the germination rates were calculated, and the fresh weights of the seedlings were determined to evaluate the growth.

To evaluate the long-term impact on plant growth, soil samples containing PBSA residue were filled into 3-inch pots (300 g soil/pot). Each group contained 8–10 seedlings of Chinese cabbage, which were grown in potting soil for 7 days and transplanted into each pot. The rate of fertilizer application followed that of a previous study [46]. During the cultivation period, 0.05 g chemical fertilizer (Sinon Chemical Industry Co., Ltd., Taichung, Taiwan, with an N:P:K ratio of 14:15:10) was applied every week. After 21 days of cultivation, the shoot fresh and dry weights of the plants at five-leaf ages were measured.

## 3. Results

### 3.1. Isolation and Identification of Two Elite PBSA-Degrading Fungal Strains

We applied a rapid screening platform (i.e., clear zone method) to screen and isolate PBSA-degrading microorganisms from soil samples in this study. As shown in Figure S1, several transparent circles with different sizes were formed on the PBSA agar plates. We noticed that the numbers of clear zones varied with sampling location. Among the samples, those derived from the tomato field located in Xiushui township (CHT) and the paddy field in Hsinchu (HCP-A) showed better degrade abilities than those collected in other places. It was previously shown that microbial degradation is associated with temperature [47]. We found that the PBSA degradation rates were elevated at relatively high temperatures. This phenomenon was observed among the samples of the deep compost soil at National Taiwan University (NTUCS-D), the paddy fields in Hsinchu (HCP-A, B), and site D of the Xindian riverside (Xindian D). When clear zones were formed on the plates, a single colony of individual PBSA-degrading microorganisms was isolated by the streaking plate method.

**Figure 1:**
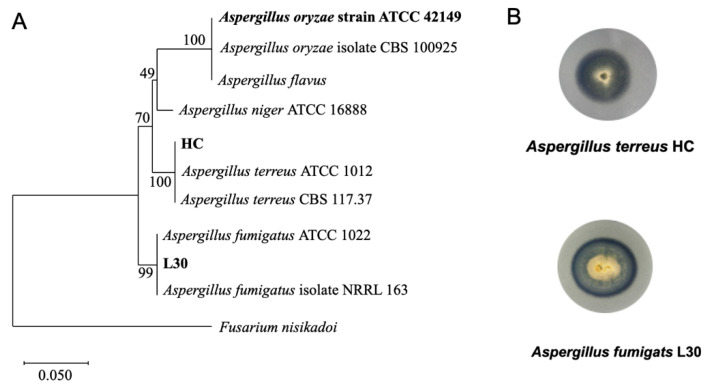
Phylogenetic tree of ITS and clear zone formation on PBSA agar plates. (**A**) A phylogenetic tree of two selected fungal strains (*Aspergillus terreus* HC and *Aspergillus fumigatus* L30) was constructed from a comparison of sequences of approximately 400 bp of the internal transcribed spacer (ITS) region using neighbor-joining analysis of a distance matrix with Kimura’s two-parameter model. Bootstrap values (expressed as percentages of 1000 replications) greater than 75% are shown at branch points. A reference fungal strain, *Aspergillus oryzae* RIB40 (ATCC42149), was used as a control in this study, and *Fusarium nisikadoi* was used as an outgroup. The scale bar represents 0.05 substitutions per nucleotide position. (**B**) Morphology of the elite PBSA-degrading fungal strain.

According to the morphological characteristics of the isolates with hyphae and spores, we assumed that these isolates were fungi. We isolated dozens of fungal strains from the soil sampling sites, eight of which showed obvious PBSA-degrading activities and were sequenced (Figure S2). Two strains, L30 and HC, were selected based on their growth rates and clear zone sizes. These two strains were derived from a lemon field located in Chaozhou township, Pingtung County, and the B side of a paddy field in Hsinchu. L30 and HC showed high sequence similarity with *Aspergillus fumigatus* (99.74%) and *Aspergillus terreus* (99.78%) by the partial sequences of ITS sequences, respectively. A phylogenetic tree was built from the partial sequences of ITS to elucidate the taxonomic position of these strains (Figure 1A). *A. oryzae* RIB40 (ATCC42149) was used as a positive control for PBSA degradation tests in this study [48]. The clear zones as well as the morphologies of these strains formed on PBSA agar plates are shown in Figure 1B.

### 3.2. Two Elite Fungal Strains Showed High PBSA Biodegradation Efficacy

**Figure 2:**
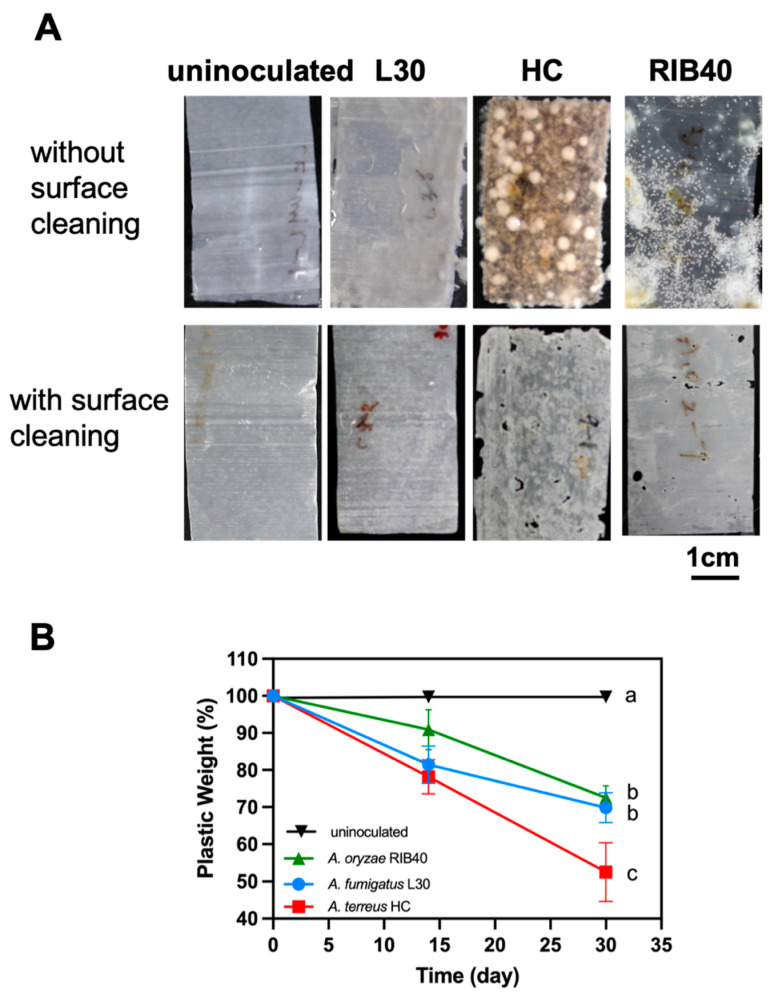
Colonization of fungal strains on PBSA plastic films and their degradation abilities. (**A**) Biodegradation PBSA films after 30 days of incubation. Twelve pieces of PBSA films (size: 2.5 × 5.0 cm^2^; thickness: 50 μm) were cocultured with *A. terreus* HC, *A. fumigatus* L30, and *A. oryzae* RIB40 (ATCC42149) at 30 °C in a carbon-free basal medium. The upper panel shows the attached hyphae of individual strains on the surface of PBSA plastic films after 30 days of incubation. The bottom panel shows the surface of plastic films after hyphae removal by rinsing with DDW. (**B**) Weight loss of PBSA plastic film against time. The sampling times were 0, 14, 30 days after incubation. The values are expressed as the mean ± standard deviation of three biological replicates (*p* < 0.05; Tukey’s post hoc ANOVA test).

After coincubation with individual elite fungal strains and PBSA film in a carbon-free basal medium for 30 days, hyphae were observed on the surface of the plastic film (Figure 2A, upper panel). After removing the hyphae, several cracks and cavities were observed on the surface of the plastic films treated with *A. terreus* HC or *A. oryzae* ATCC42149 (Figure 2A, bottom panel). On the other hand, the films treated with *A. fumigatus* L30 became relatively thin and transparent in comparison with untreated films (Figure 2A, bottom panel). The degradation rates of individual elite fungal strains were determined by the weight loss (WL) of the films. As shown in Figure 2B, the weights of the plastic films were significantly reduced by biodegradation. The WL of the plastic films treated with *A. terreus* strain HC, *A. fumigatus* strain L30, and *A. oryzae* RIB40 for 30 days were 47.5%, 30.2%, and 27.5%, respectively. Since *A. terreus* HC showed better degrading ability than the other strains, we used this strain for subsequent soil burial tests.

### 3.3. Effect of Replacement or Addition of Fresh Medium on PBSA Plastic Film Degradation

**Figure 3:**
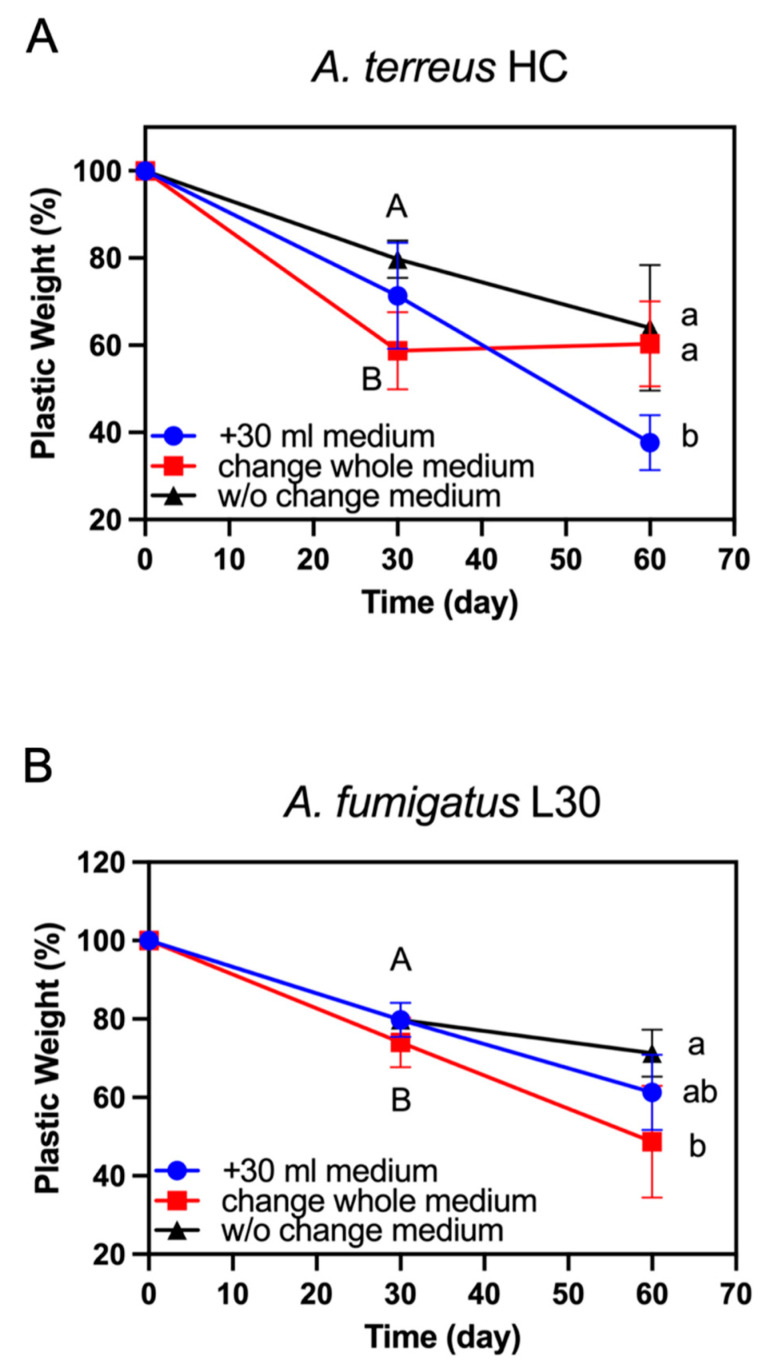
Determination of the effect of replacement or addition of fresh medium on PBSA plastic film degradation after 60 days of incubation. (**A**) Weight loss of PBSA film against time with *A. fumigatus* strain L30. (**B**) Weight loss of PBSA film against time with *A. terreus* HC. w/o medium change: maintaining the original medium during the whole incubation period (60 days); replacement of all culture medium (100 mL) or addition of 30 mL of fresh medium were carried out on the 30th day of incubation. The value of individual treatment is expressed as the row mean ± standard deviation. Statistical analyses were based on the degradation rates (g/day) on the 30th (in uppercase letters A and B) and 60th (in lowercase letters a and b) days of incubation.

To evaluate the degradation rates of the elite strains over a relatively long period of time, we conducted a degradation experiment after 60 days of incubation. As shown in Figure 3, the degradation rate of *A. fumigatus* strain L30 or *A. terreus* strain HC decreased after 30 days of incubation, and the latter was obviously changed compared with the former. We deduced that nutrient depletion occurred during this incubation period. Accordingly, we evaluated two strategies to rescue the degradation rates. One strategy was to add fresh medium (30 mL), and the other strategy was to replace all of the old medium (100 mL) after 30 days of incubation. In comparison with those of medium without change, we found that the degradation rate of either *A. fumigatus* strain L30 (Figure 3A) or *A. terreus* strain HC (Figure 3B) was increased when 30 mL of fresh medium was added into the culture fluid after 30 days of incubation, although there was no significant difference in the degradation ability of the former strain. The degradation rates of these strains on the 60th day remained almost the same as those before 30 days of incubation. For the trials to replace the whole old culture broth (100 mL), there were remarkable differences between the two strains. As shown in Figure 3A, the degradation rate of *A. fumigatus* strain L30 was improved significantly in comparison with that of strain in the medium that was unchanged after 30 days of incubation. In contrast, the degradation rate of *A. terreus* strain HC was almost reduced to zero after replacing the old incubation broth with a fresh medium (Figure 3B).

### 3.4. Appearance of PBSA Plastic Films Degraded by Two Elite Aspergillus Strains

**Figure 4:**
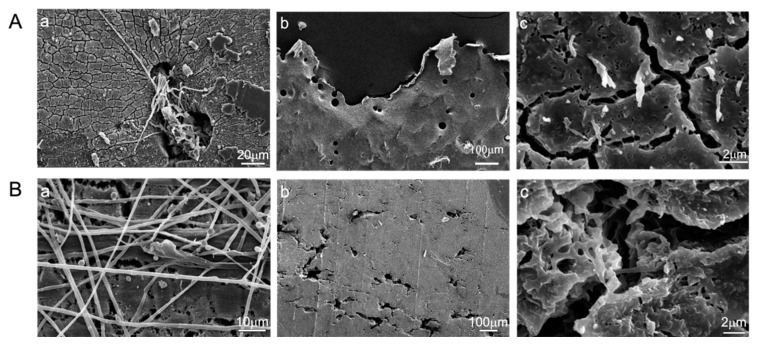
Scanning electron microscopic images of plastic films degraded by two elite fungal strains. PBSA films degraded by *A. terreus fumigatus* L30 (**A**) and HC (**B**) after 30 days of incubation. (**Aa**) The attachment and network of *A. terreus* L30 hyphae on the surface of PBSA film, (**Ab**) cracks and holes on the plastic surface, (**Ac**) irregular cavity in internal of plastic film. (**Ba**) *A. fumigatus* HC hyphae intertwined inside the plastic film and formed holes on the plastic surface, (**Bb**) cracks and holes on the plastic surface, and (**Bc**) internal erosion of the plastic film.

To observe erosion or decomposition of the plastic surface by elite strains after 30 days of incubation, SEM analysis was conducted. As shown in Figure 4 and Figure S3, the SEM images showed a PBSA film with an intact surface when not inoculated with *Aspergillus* (Figure S3). When inoculated with *Aspergillus* strains, filamentous hyphae of *A. fumigatus* L30 or *A. terreus* HC densely covered the surface and even intertwined inside and penetrated the plastic film, as shown in Figure 4(Aa,Ba). While removing the hyphae, many cracks and holes were observed on the film surface (Figure 4(Ab,Bb). Moreover, internal erosion also occurred, as shown in Figure 4(Ac,Bc), and irregular cavities were observed inside the PBSA films. The visual results suggest that biodegradation occurred not only on the surface of PBSA films but also inside the films.

### 3.5. Changes in the Chemical Composition of PBSA Films during Biodegradation

**Figure 5:**
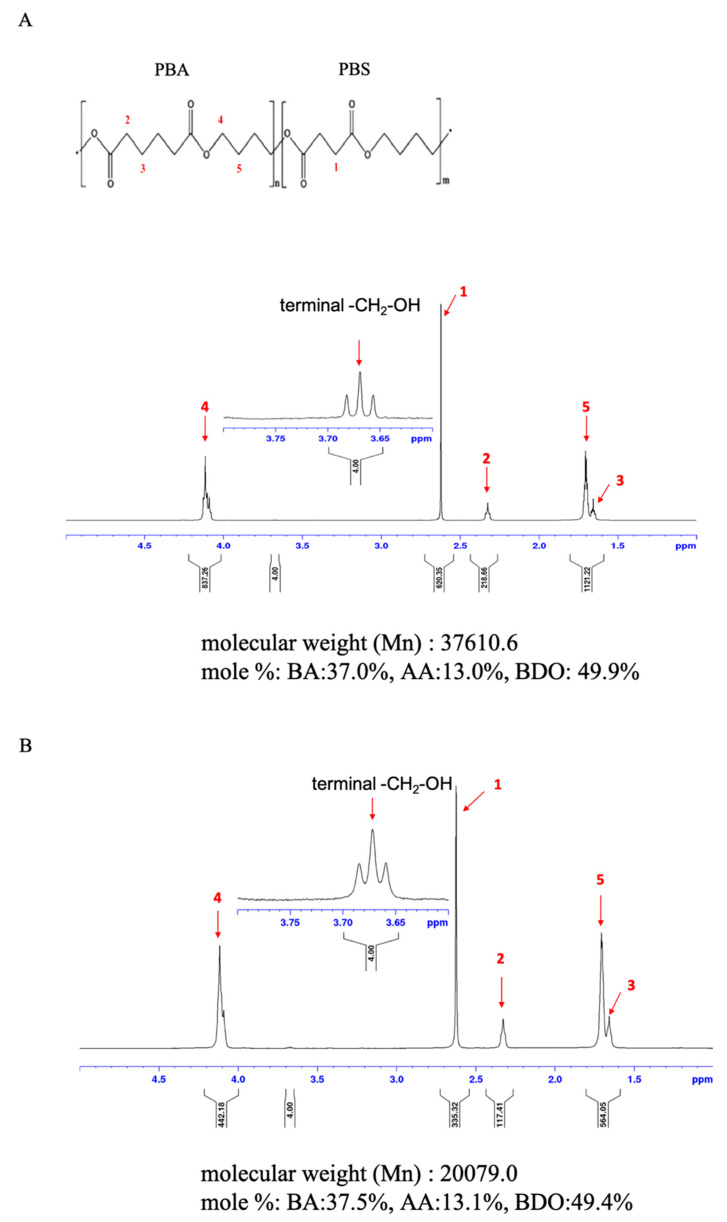
NMR spectra of PBSA films after degradation by *A. terreus* HC. (**A**) PBSA film without degradation. (**B**) PBSA film degraded by *A. terreus* HC for 30 days. Compared with the original PBSA film, the PBSA film, after 30 days of biodegradation, showed a lower number average molecular weight (Mn) but a similar monomer composition ratio. Peaks 1–5 correspond to the main signals of protons shown in (**A**,**B**). Peak 1 represents succinic acid (SA), peaks 2 and 3 represent adipic acid (AA), and peaks 4 and 5 represent 1,4-butanediol (BDO).

To confirm the chemical composition of PBSA films before and after biodegradation, we conducted an NMR analysis. The ^1^ H-NMR spectrum of PBSA is shown in Figure 5 with a chemical structure illustration. The peaks marked with 1, 2, 3, 4, and 5 (Figure 5) indicated protons of SA (1), AA (2, 3), and BDO (4, 5). We compared the intensity of terminal H moieties with the five main signals, and the intensity ratios of the PBSA film without degradation were 4.00:620.35 (1), 4.00:218.66 (2), 4.00:1121.22 (3 and 5), and 4.00:837.26 (4) (Figure 5A). After biodegradation with *A. terreus* HC for 30 days, lower intensity ratios were shown as 4.00:335.32 (1), 4.00:117.41 (2), 4.00:564.05 (3 and 5), and 4.00:442.18 (4) (Figure 5B). As shown in Figure 5, the Mn value decreased from 37610.6 to 20079.0. The weakening of the relative intensity ratios and the decrease in the Mn suggested that structural weakening occurred in the PBSA films due to decomposition by *A. terreus* HC.

### 3.6. Biodegradation of PBSA Films in Farmland Soil

**Figure 6:**
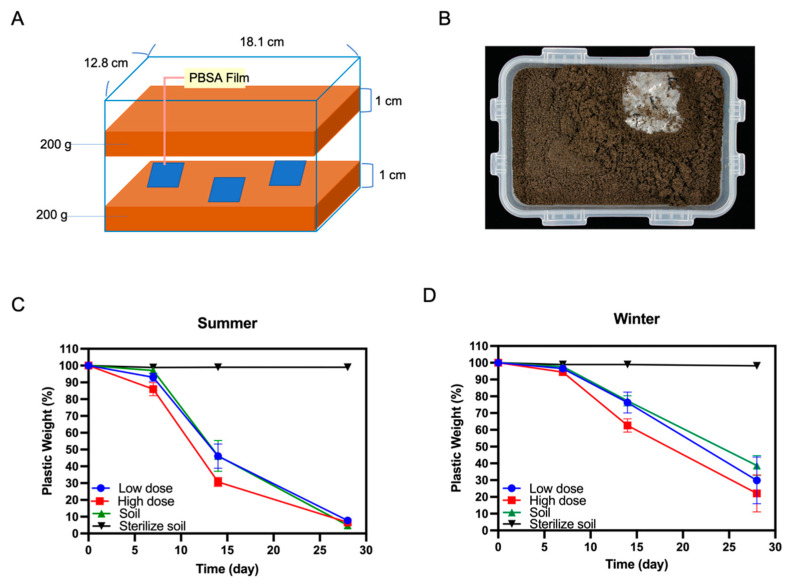
Soil burial test for biodegradation of PBSA films. (**A**) Schematic diagram of the device for the soil burial test. (**B**) A piece of degraded PBSA film before analysis. (**C**) Biodegradation of PBSA films buried in summer soil. (**D**) Biodegradation of PBSA films buried in winter soil. PBSA plastic films were buried in NTU farmland soil that was collected from late summer (September) or winter (December) in 2020. Low (10 mL of mycelial suspension) or high (50 mL of mycelial suspension) doses of *A. terreus* HC fungal mycelia were inoculated into the soil. The values for the respective weights of PBSA film are the mean ± standard deviation of triplicate samples (*p* < 0.05; Tukey’s post hoc ANOVA test).

To simulate the degradation of PBSA films under general soil environments, we conducted a soil burial test (Figure 6A,B). Soil samples were collected from NTU farmland in September (late summer) and December (winter) of 2020 to evaluate the effect of seasonal conditions on biodegradation. The PBSA films retained approximately 98% of their weight after being buried in sterile soil for 28 days (Figure 6B,C). The PBSA films were degraded by approximately 95% while buried in the nonsterile soil collected in September after 28 days of incubation (Figure 6C), whereas the films were degraded by 61.3% when buried in soil collected in December (Figure 6). We evaluated the effects of supplementation with different doses of *A. terreus* HC fungal mycelia on degradation rates. For the soil collected in September, the weights of PBSA were reduced by 54.9% and 69.3% when the nonsterile soil was supplemented with low and high doses of fungal mycelia, respectively, after 14 days of incubation (Figure 6C). The film weights achieved 92.2% and 93.4% reductions after 28 days of degradation at low and high fungal mycelia doses, respectively (Figure 6C). For the soil collected in December, the weights of PBSA were reduced by 23.7% and 37.4% when the nonsterile soil was supplemented with low and high fungal mycelia doses, respectively, after 14 days of incubation (Figure 6D and Figure S4A). This result indicated that the addition of a high dose of *A. terreus* HC fungal mycelia to winter soil could improve the degradability (+14%) in the early stage of incubation (14 days). After 28 days of incubation, the weight of PBSA films was reduced by 70.2% and 78% with low and high fungal mycelia doses, respectively, and there was no significant difference between the two treatment groups (Figure 6C).

### 3.7. Lipolytic Enzyme Activities of A. terreus HC and Soil

**Figure 7:**
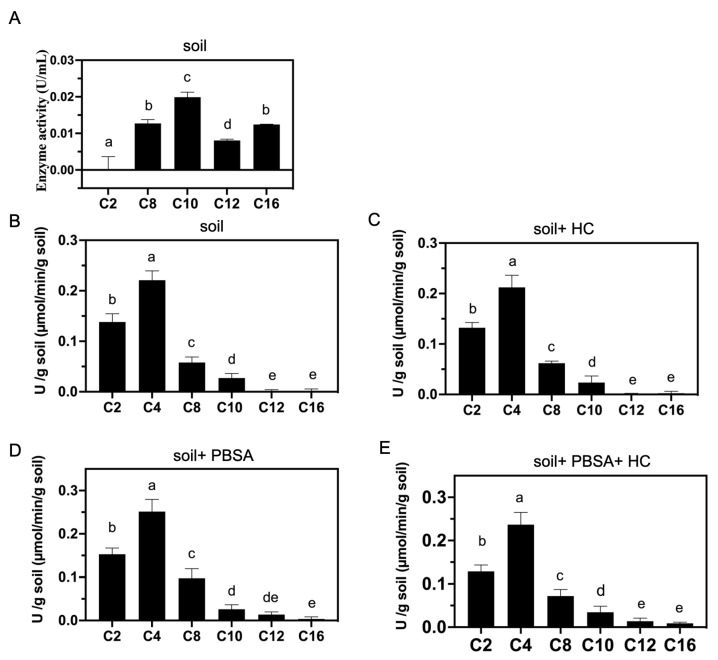
Lipolytic enzyme activities in the *A. terreus* HC culture broth and soil. (**A**) The lipolytic enzyme activities of *A. terreus* HC in the culture broth were determined by chromogenic nitrophenyl esters with different chain lengths as substrates (i.e., *p*-nitrophenyl esters). The supernatant of the *A. terreus* HC culture was collected from the culture fluid incubated with PBSA film and *A. terreus* HC for 60 days. (**B**) The lipolytic enzyme activities of soil. (**C**) The lipolytic enzyme activities of soil supply with *A. terreus* HC. (**D**) The lipolytic enzyme activities of soil buried with PBSA. (**E**) The lipolytic enzyme activities of PBSA buried soil supply with *A. terreus* HC. The assay was conducted after 30 days of PBSA degradation in soil. The substrates used were as follows: C2, 4-nitrophenyl acetate; C4, 4-nitrophenyl butyrate; C8, 4-nitrophenyl caprylate; C10, 4-nitrophenyl decanoate; C12, 4-nitrophenyl dodecanoate; and C16, 4-nitrophenyl palmitate. The results are presented as the mean ± standard deviation (*p* < 0.05; Tukey’s post hoc ANOVA test).

We extracted crude enzymes from the supernatant of *A. terreus* HC broth after 60 days of incubation in the presence of PBSA films. Lipolytic enzymes included lipases and esterases. We used *p*-nitrophenyl fatty acid esters with different chain lengths (C2, C4, C8, C10, C12, and C16) as substrates to investigate the substrate specificity of the potential lipolytic enzymes of *A. terreus* HC. As shown in Figure 7A, the lipolytic enzymes produced by *A. terreus* HC displayed relatively high activities for C8 to C16 chain fatty acids (i.e., medium to long-chain fatty acids), and the maximal activity was observed for C10 fatty acids. Intriguingly, we noticed that although the *p*-nitrophenyl ester with a short chain (i.e., 4-nitrophenyl acetate, C2) has often been selected as a candidate substrate for lipolytic enzyme activity assays in the literature, it was not detected in the extracellular medium of *A. terreus* HC after PBSA degradation (Figure 7A).

After conducting the soil burial test (Figure 7B–E), we further determined the lipolytic enzyme activities of the soil samples (NTU farmland) with the same substrates mentioned above. As shown in Figure S5A, lipolytic enzyme activities of the test soil were detected against all the substrates of C2 to C16 chain fatty acids. We found that the activities were relatively high against those of short-chain fatty acids (C2 to C4) and decreased with increasing carbon chain length (C8 to C16). When PBSA films were buried in the soil for 30 days, we found that the lipolytic enzyme activities against the substrates with C8 and C12 chain lengths were remarkably higher than those in soil without PBSA films (Figure S5B and Figure 7D,E). Furthermore, we noticed that when a suspension of *A. terreus* HC mycelia was added to the test soil at the beginning of the experiment, the lipolytic enzyme activities against the substrates with C10 and C16 chain lengths were dramatically higher than those without fungal addition, which were 1.3- and 5.8-fold increased, respectively (Figure S5B).

### 3.8. Phytotoxicity Assessment

**Figure 8:**
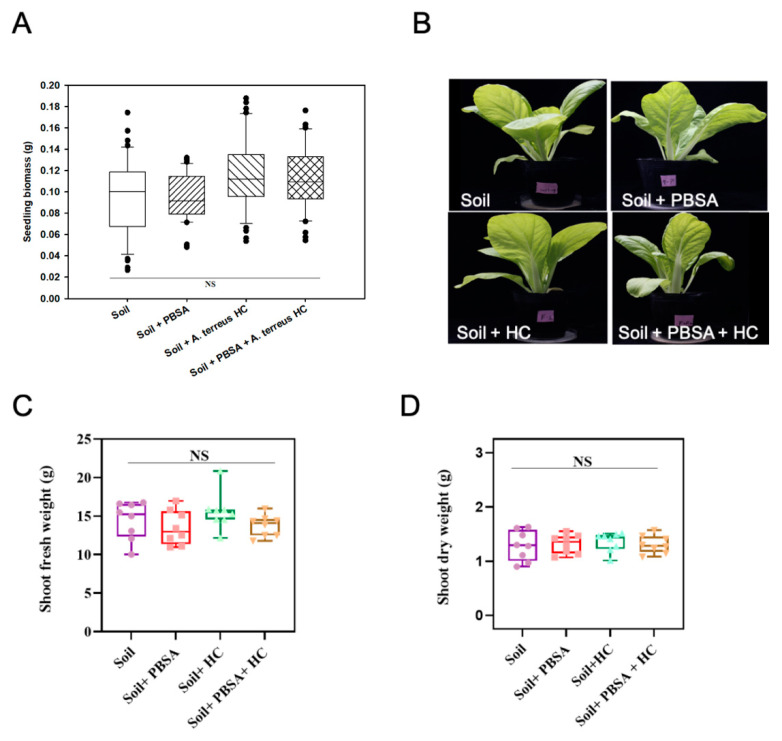
Phytotoxicity assessment of PBSA plastic film residues or *A. terreus* HC on plant growth. Seeds of Chinese cabbage were cultivated in soil containing 30-day degraded PBSA film residues to determine the seedling vigor for assessing phytotoxicity. (**A**) The biomass of Chinese cabbage seedlings was assessed after 8 days of germination. (**B**) The phenotype of Chinese cabbage at 21 days of cultivation. (**C**,**D**) Fresh and dry weights of shoots after 21 days of cultivation in soil containing PBSA residues. Ns: no significant difference among the treatments. The results are presented as the mean ± standard deviation (*p* < 0.05; Tukey’s post hoc ANOVA test).

Phytotoxicity assessment was conducted to evaluate whether there were potential adverse effects derived from the soil containing PBSA residues or *A. terreus* HC on the agricultural environment or plant growth. Chinese cabbage, a popular green leafy vegetable in many Asian countries, was used as the test material. As shown in Figure 8, the germination rates in each treatment group were not significantly different and were 55%, 58.8%, 57.5%, and 60% for the soil, soil + PBSA, soil+ *A. terreus* HC, and soil + PBSA+ *A. terreus* HC treatments, respectively, and the average biomass of the seedlings did not significantly vary, with values of 0.10 g, 0.11 g, 0.12 g, and 0.11 g, respectively (Figure 8A).

For the assessment of adult plants, we found no significant difference among the treatments either in morphology or in biomass (shoot fresh and dry weight) of Chinese cabbage after 21 days of cultivation in the PBSA-containing soil (Figure 8B–D). The average fresh weights were 14.44, 13.51, 15.60, and 13.77 (g), and the dry weights were 1.29, 1.32, 1.35, and 1.31 (g) for the soil, soil + PBSA, soil+ *A. terreus* HC, and soil + PBSA+ *A. terreus* HC treatments, respectively. These results indicated that neither PBSA residue nor *A. terreus* HC influenced the germination rate, seedling biomass, and growth of the test green leafy vegetable.

## 4. Discussion

### 4.1. Individual Aspergillus Strains Showed Unique PBSA Degradation Characteristics

In this study, two PBSA-degrading *Aspergillus* strains (*A. terreus* HC and *A. fumigatus* L30) were isolated from farmland soil in Taiwan. As shown in Figure 3, the degradation rate of *A. fumigatus* L30 or *A. terreus* HC was increased, while 30 mL of fresh basal medium was added to the incubation broth after 30 days of incubation (i.e., the blue curves). Some literature mentioned that supplementation with other nutrients or substrates can stimulate the degradation ability, which may be due to increased microbial biofilm growth or the provision of an easily metabolized nutrient source for microbes [49,50,51,52]. We deduced that this advantageous effect was due to an increase in cell growth and promotion of the synthesis of degrading enzymes by adding fresh basal medium during fed-batch cultivation.

On the other hand, we noticed that while the whole suspension (100 mL) of cell culture was removed and replaced with the equivalent amount of fresh basal medium, the PBSA degradation (30–60 days) of *A. fumigatus* L30 was not affected (i.e., the red curve in Figure 3A), whereas that of *A. terreus* HC was almost terminated (i.e., the red curve in Figure 3B). The case of *A. terreus* HC was reminiscent of the phenomenon previously reported by Bottone and colleagues [53]. They found that some filamentous fungi, such as *Mucor* and *Aspergillus* spp., the inhibitory substances were self-produced during their culture under minimal medium, which precluded the new hyphal backgrowth in the fresh medium. They also observed that even inoculation with fresh spore suspension did not result in new hyphal growth. These inhibitory substances could be bicarbonate, which was identified in the respiratory metabolism of *Aspergillus nidulans* [54,55]. Accordingly, we assumed that *A. terreus* HC produced the still unidentified substance to inhibit its metabolic activity while the whole culture suspension was replaced by a fresh basal medium, although this remains to be elucidated.

### 4.2. PBSA Degradation Efficiency of the Winter Soil Can Be Improved by Addition of Fungal Mycelia

To evaluate the effect of temperature on PBSA degradation, we sampled the farmland soil in different seasons (September and December) for the soil burial test. As shown in Figure 6, a higher degradation rate was shown in the treatment using late summer (September) soil than that using early winter (December) soil. Hoshino and colleagues proposed PBSA degradation rates under different weather conditions, which were correlated with the accumulation of daily effective temperatures (i.e., effective temperature = T−10 °C, T: daily temperature) [56]. We deduced that increasing the temperature increases the rate of effective biodegradation by microbes in the soil sample. As mentioned above, the degradation activity of the indigenous winter soil was lower than that of the summer soil (Figure 6). We found that adding a high dose (50 mL of mycelial suspension) of *A. terreus* HC mycelia to the winter soil sample could improve the degradation rate by 15% at day 14. Such an effect of bioaugmentation with elite microbial inoculant was also reported in previous studies. For example, Ishii and colleagues reported that the addition of 10 mg of wet cells of *A. fumigatus* NKCM1706 into the soil could promote sixfold PBS degradation at 30 °C [57]. On the other hand, such a bioaugmentation effect was not observed in the trial using the summer (September) soil sample (Figure 6C). The degradation rate of the September soil group without fungal mycelia inoculation was relatively high on day 14 of incubation, suggesting that indigenous microbes already possessed high degradation activities. Accordingly, even supplementation with a high dose of *A. terreus* HC mycelia did not exceed the background effect. Taken together, these results suggest that supplementation with proper amounts of elite microbial cultures, such as *A. terreus* HC mycelia, is a practical strategy to enhance PBSA biodegradation activity in environments with poor decomposition.

### 4.3. PBSA Could Induce the of Lipolytic Enzyme Activities in Farmland Soil

The lipolytic enzyme activities of the NTU farmland soil samples were determined and shown in Figure 7. We found that the soil showed lipolytic activities against all the substrates of C2 to C10 chain fatty acids even in the absence of PBSA film. When PBSA films were buried in the soil for 30 days, the activities were significantly increased against the substrates with C8 and C12 chain lengths compared with the activities without PBSA addition (Figure 7D). This result indicated that PBSA induced the synthesis of lipolytic enzymes of indigenous microbes to degrade substrates with medium and long carbon chains in soil. In a previous study, Yamamoto-Tamura and colleagues observed a similar phenomenon [16]. The authors reported that the activity of soil esterase was remarkably induced upon the addition of PBSA film. Accordingly, we assumed that the presence of PBSA induced esterase production from indigenous microbes in the soil. Moreover, we noticed that when *A. terreus* HC mycelia were added to the soil with PBSA films (i.e., soil + PBSA + HC) (Figure 7E), the lipolytic enzyme activities against the substrates with C10 and C16 chain lengths were further increased in comparison with those without the fungal supplement (i.e., soil + PBSA) (Figure 7D and Figure S5). Taken together, these results, with respect to soil lipolytic enzyme activities, showed that the NTU farmland soil mainly contains short-carbon-chain enzymes, but when the soil contained buried PBSA films, indigenous soil microorganisms were triggered to secrete long carbon chain-degrading enzymes. Moreover, while adding plastic decomposing microbes can further promote the secretion of long carbon chain-degrading enzymes, we deduced that the elite fungal strain *A. terreus* HC can degrade PBSA into shorter-chain fatty acids, which are then catabolized by the other indigenous microorganisms in the soil. This hypothesis remains to be proved by further analysis.

### 4.4. No Observed Adverse Effect on the Growth of Leafy Vegetable by PBSA Degradation or the Addition of Elite Fungal Culture in Farmland Soil

Plastic pollution has become a global concern for ecosystem health and biodiversity conservation. People are increasingly interested in understanding the impact of biodegradable plastic on ecosystems [4,5,58,59]. The analysis of the toxicity of biodegradable mulch to plants is mainly carried out by measuring plant growth in soil containing biodegradable plastic film fragments [60,61,62,63,64]. Since the sensitivity of plants to a variety of biodegradable materials is species dependent, a few studies have shown that some products may alter plant development, while a few others showed certain mulches to be likely safer for use in agricultural environments [4]. Wang and colleagues reported that soil containing the residue of PBS-based copolymers (P(BS-co-SA)) did not affect the growth of Chinese cabbage [6]. Furthermore, approximately 0.6~2% (*w*/*w*) of PLA, PHB, PBAT, or the innovative biodegradable material Mater-Bi^®^ was individually buried in the testing soils for 6–7 months, and plant seeds were sown inside. These studies reported that there was no adverse effect on either the germination rates of cress, barley s, Brassica, and sorghum or the dry weight of seedlings [61,63,64]. On the other hand, Fritz and colleagues found that when the soil contained 2% (*w*/*w*) polyester amide mulch film fragments, the plant biomass decreased 20–50% in cress, millet, and rapeseed [60]. In this study, we conducted a phytotoxicity assessment to evaluate whether there were potential adverse effects derived from soil containing 0.2% PBSA films or that supplemented with *A. terreus* HC on plant growth. As shown in Figure 8, we did not observe an obvious adverse influence of PBSA degradation or the addition of fungal culture on the seed germination rate, seedling, and mature plant weight of Chinese cabbage. Whether PBSA or *A. terreus* HC in soil affects other kinds of plants, soil microbial communities, or other organisms requires further research.

## 5. Conclusions

In this study, we selected two elite PBSA-degrading *Aspergillus* strains, namely, *A. terreus* HC and *A. fumigatus* L30, from soil samples in Taiwan. It is noteworthy that biodegradation of PBSA by *A. terreus* is reported for the first time. The PBSA films placed in the carbon-free basal medium were approximately 42% degraded by *A. terreus* HC within 30 days of incubation, while the films were 26% degraded by *A. fumigatus* L30. In the soil burial test, *A. terreus* HC showed over 90% and 75% degradation rates for summer and winter soil environments, respectively. When adding a high dose of *A. terreus* HC mycelia to the winter soil, the degradation efficacy of PBSA was further improved. This suggests that the application of an elite fungal mycelial suspension to soil with a low degrading ability can aid in its degradability. According to the results of the soil burial test, it can be deduced that PBSA can induce the synthesis of lipolytic enzymes of indigenous microbes to degrade substrates with medium and long carbon chains in the soil. In the phytotoxicity test, the degradation of PBSA films or the addition of fungal mycelia did not cause obvious adverse effects on the growth of seedlings or adult plants of Chinese cabbage. Taken together, the results of this study not only advance our understanding of the biodegradation of PBSA by elite *Aspergillus* strains but also provide insight into improving the efficiency of biodegradation in soil environments with moderate temperature.

## Data Availability

The data presented in this study are available on request from the corresponding author.

## Supplementary Materials

The following supporting information can be downloaded at: https://www.mdpi.com/article/10.3390/polym14071320/s1, Figure S1. Clear zones formed by PBSA-degrading microorganisms derived from different sampling sites. Figure S2. Morphology of the respective elite PBSA-degrading fungal strain. Figure S3. Scanning electron microscopy of an uninoculated PBSA plastic film. Figure S4. Weight loss (%) of PBSA plastic films against degradation time in soil burial tests. Figure S5. Lipolytic enzyme activities in the soil.
