# Supplementary material for: Biodegradation of PBSA Films by Elite Aspergillus Isolates and Farmland Soil
Source: Polymers (Basel). 2022 Mar 24;14(7):1320. doi: 10.3390/polym14071320 (PMC9002719; doi:10.3390/polym14071320)
Supplement: Supplementary file 1 [file polymers-14-01320-s001.zip › polymers-1617490-supplementary.pdf]

polymers

MDPI

Biodegradation of PBSA Films by Elite *Aspergillus* Isolates and Farmland Soil

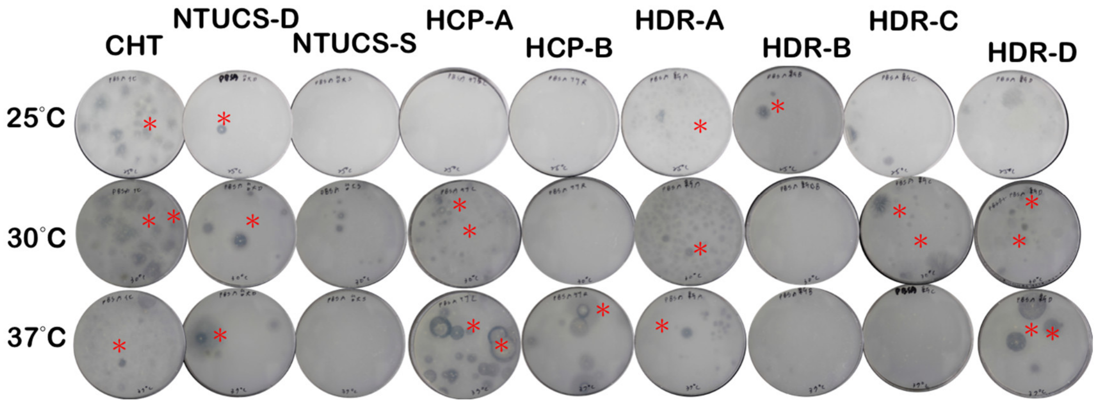

Figure S1. Clear zones formed by PBSA-degrading microorganisms derived from different sampling sites. The test soils were collected from the following 8 sites (9 samples in total) in Taiwan. The experiments were conducted at 25, 30, and 37°C. The CHT sample was collected from a tomato farm located in Xiushui Township, Changhua County. NTUCS-S and NTUCS-D samples were collected from a composting yard at National Taiwan University. HCP-A and HCP-B samples were collected from a rice paddy field in Xinfeng Township, Hsinchu County. XDR-A to XDR-D samples were collected from riverside banks along the Xindian River. All the soil samples were sieved through a 3 mm mesh before use. Asterisks mark clear zones formed by PBSA-degrading microorganisms.

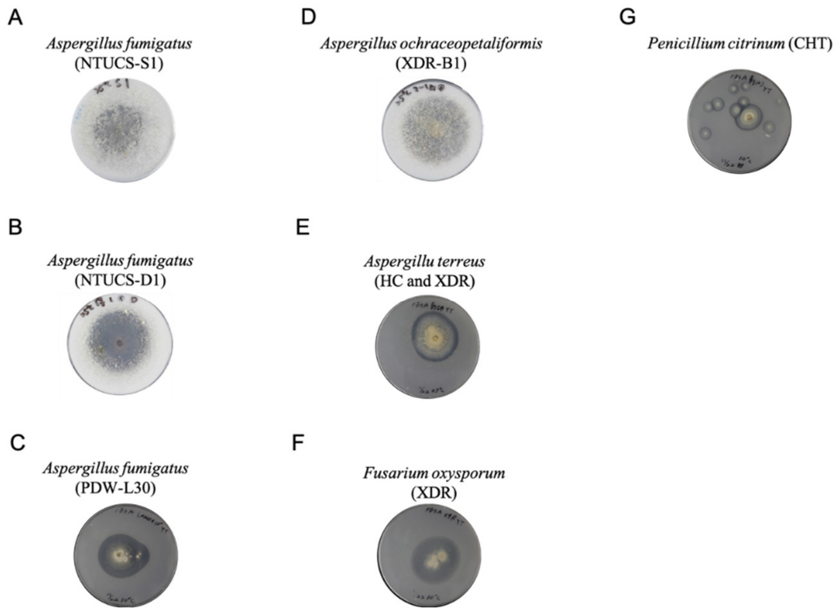

Figure S2. Morphology of the respective elite PBSA-degrading fungal strain. (**A**) NTUCS-S1: collected from the surface (0-20 cm depth) of a composting yard at National Taiwan University. (**B**) NTUCS-D: collected at a 50 cm depth in a composting yard at National Taiwan University. (**C**) PDW-L30: collected from lemon orchard soil located in Chaozhou Township, Pingtung County. (**D**) XDR-B1: Xindian riverbank. (**E**) Fungal isolates derived from a rice paddy field in Xinfeng Township, Hsinchu County (HC) and the Xindian riverbank (XDR). (**F**) XDR: Xindian riverbank. (G) CHT: a tomato farm located in Xiushui Township, Changhua County.

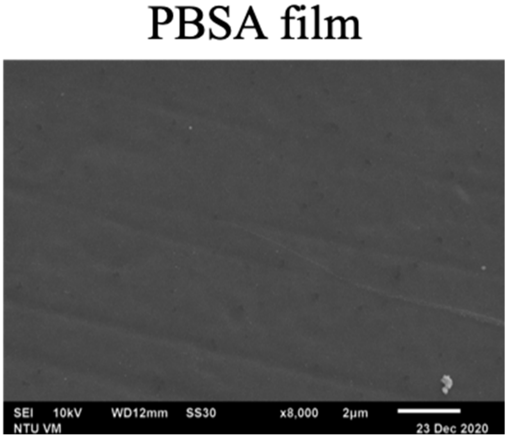

Figure S3. Scanning electron microscopy of an uninoculated PBSA plastic film.

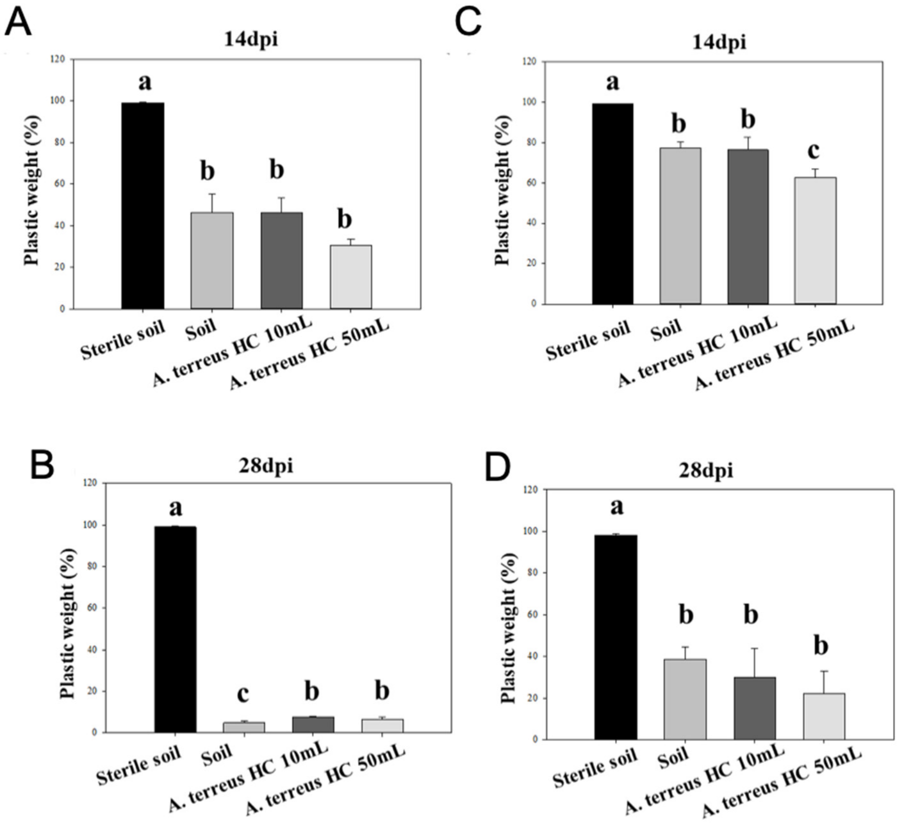

Figure S4. Weight loss (%) of PBSA plastic films against degradation time in soil burial tests. (**A**) Plastic weight after 14 days of degradation in summer soil. (**B**) Plastic weight after 28 days of degradation in summer soil. (**C**) Plastic weight after 14 days of degradation in winter soil samples. (**D**) Plastic weight after 28 days of degradation in winter soil samples.

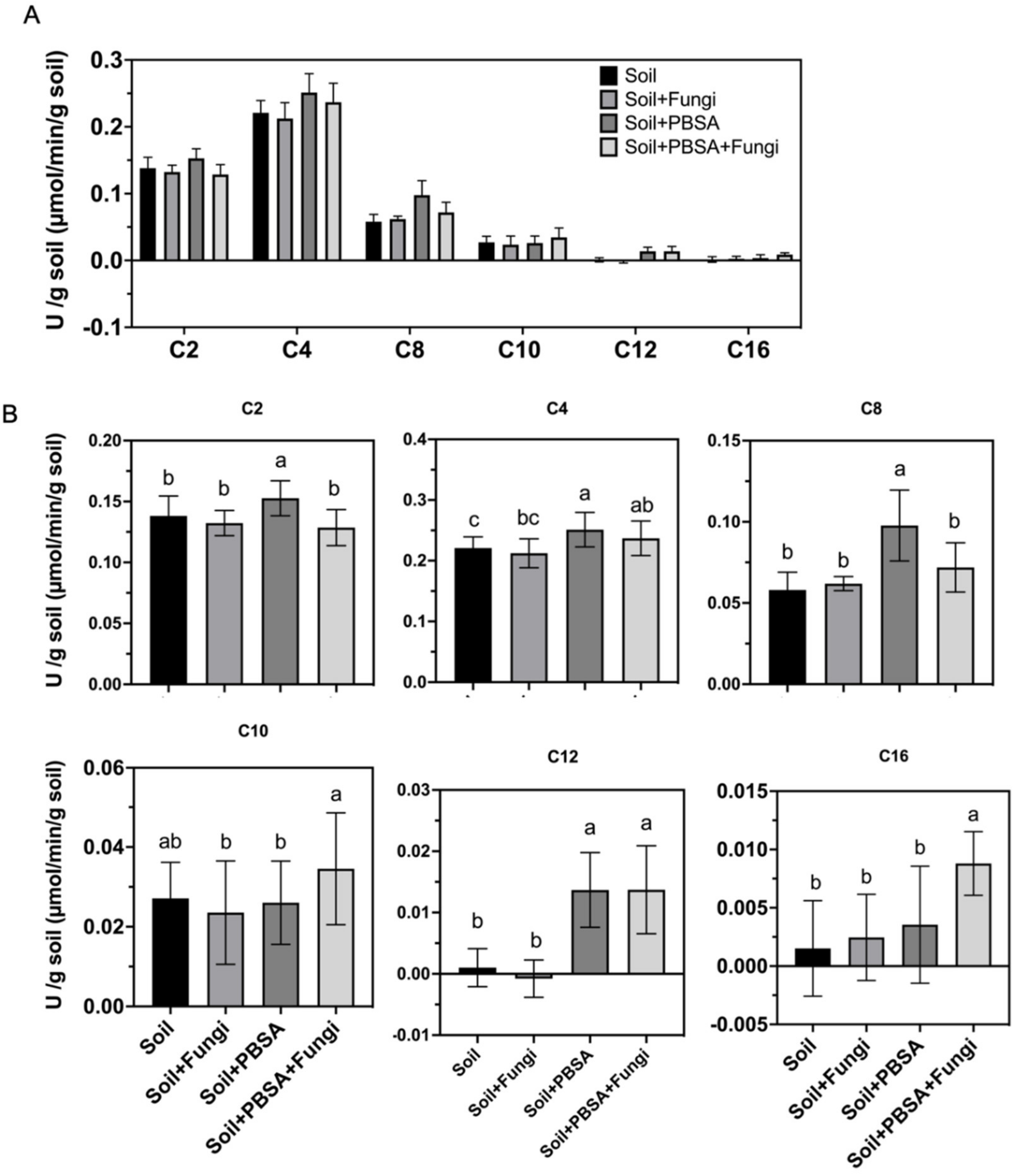

Figure S5. Lipolytic enzyme activities in the soil. (**A**) The lipolytic enzyme activities of *A. terreus* HC in the culture broth were determined by chromogenic nitrophenyl esters with different chain lengths as substrates (i.e., p-nitrophenyl esters). The supernatant of the *A. terreus* HC culture was collected from the culture fluid incubated with PBSA film and *A. terreus* HC for 60 days. (**B**) The lipolytic enzyme activities of different substrates. The substrates used were as follows: C2, 4-nitrophenyl acetate; C4, 4-nitrophenyl butyrate; C8, 4-nitrophenyl caprylate; C10, 4-nitrophenyl decanoate; C12, 4-nitrophenyl dodecanoate; and C16, 4-nitrophenyl palmitate. The assay was conducted after 30 days of PBSA degradation in soil. The results are presented as the mean ± standard deviation ($P<0.05$; Tukey's post-hoc ANOVA test).
